# Chestnut–quebracho tannins shape the fecal metabolome of weaned pigs through microbiota-dependent urolithin production

**DOI:** 10.1186/s40104-026-01503-x

**Published:** 2026-10-06

**Authors:** Federico Correa, Diana Luise, Francesco Palumbo, Daniel Scicchitano, Simone Rampelli, Silvia Molino, Nicola Panciroli, Marco Candela, Beatriz Garay-Mayol, María Ángeles Ávila-Gálvez, Antonio González-Sarrías, Andrea Castagnetti, Paolo Trevisi

**Affiliations:** 1 Department of Agro-Food Sciences and Technologies, University of Bologna, Viale Giuseppe Fanin 46, Bologna, 40127 Italy; 2 Department of Pharmacy and Biotechnology, University of Bologna, Via Belmeloro 6, Bologna, 40126 Italy; 3 Silvateam S.p.A, Via Torre, 7, San Michele Mondovì, 12080 Italy; 4 Wellmicro S.r.l., Via Antonio Canova 30, Bologna, 40138 Italy; 5 Quality, Safety & Bioactivity of Plant Foods, Food Science & Technology Dep., CEBAS-CSIC, Murcia, 30100 Spain

**Keywords:** Gut microbiome, Metabolomics, Piglet, Post-weaning diarrhea, Tannins, Urolithins

## Abstract

**Background:**

Chestnut and quebracho tannins may support gut health in weaned piglets, but their efficacy likely depends on farm environment and microbial capacity to convert tannins into bioactive metabolites. This study evaluated the effects of a blend of chestnut-derived hydrolysable tannins and quebracho-derived condensed tannins on growth performance, diarrhea occurrence, gut metagenome, and fecal metabolome in piglets reared under two commercial farm conditions. A total of 160 weaned piglets (initial body weight 6.53 ± 0.13 kg) were assigned to a 2 × 2 factorial design comprising 2 weaning units (W1 and W2) and 2 dietary treatments (control diet or control diet + 2 g/kg tannin blend; 40 piglets per treatment × farm). The trial lasted 76 d, with weighings at T0 (entry into weaning, median 28 d), T1 (d 35), T2 (d 49), and T3 (d 76). Fecal scores and samples were collected at T1, T2, and T3 for shotgun metagenomics (155 samples per time point) and untargeted metabolomics, and targeted urolithin quantification was performed in tannin-treated piglets at T1 and T2.

**Results:**

Tannin supplementation did not affect overall growth performance throughout the weaning phase, but reduced diarrhea occurrence at T2 in W2 (*P* = 0.032). Species-level beta diversity was consistently affected by treatment across time points (*R*^*2*^= 0.01–0.03; *P* < 0.01), whereas alpha diversity was mainly farm-driven during the early post-weaning phase. Linear discriminant analysis effect size identified farm-dependent taxonomic markers, including *Escherichia coli* in control pigs from W1 at T1 and *Megasphaera elsdenii*, *Faecalibacterium prausnitzii*, and *Lactobacillus amylovorus* in tannin-treated pigs at later time points. Untargeted metabolomics revealed treatment-related fecal signatures, with isourolithin A and urolithin B among the most discriminant metabolites in treated pigs, especially in W2. Targeted analysis identified 3 urolithin metabotypes (metabotype B in 50.0% of samples), with higher isourolithin A, urolithin B, and urolithin A in W2 than W1 at T2 (*P* < 0.01). Network integration identified candidate cooperative microbial consortia associated with urolithin production, including *Ellagibacter isourolithinifaciens*; these co-abundance associations are correlative and remain to be functionally validated.

**Conclusions:**

Overall, chestnut–quebracho tannins were associated with a farm-dependent reduction in post-weaning diarrhea and modulated the gut ecosystem through microbiota-dependent polyphenol metabolism.

**Supplementary Information:**

The online version contains supplementary material available at https://doi.org/10.1186/s40104-026-01503-x.

## Background

Reducing routine antibiotic use in swine production has intensified the search for nutritional strategies that can support gut health during weaning. Weaning is a period characterized by abrupt dietary, social, and environmental stressors that predispose piglets to post-weaning diarrhea (PWD), dysbiosis, impaired barrier function, and reduced welfare [1]. Plant-derived polyphenols, and particularly tannins, have attracted growing interest as potential alternatives or complements to conventional antimicrobial approaches because of their antimicrobial, antioxidant, anti-inflammatory, and antidiarrheal properties [2]. In pigs, these effects appear especially relevant around weaning, when colonization by enterotoxigenic *Escherichia coli* (ETEC), mucosal immaturity, and instability of the gut microbiota interact to promote intestinal dysfunction [3–5]. However, the efficacy of nutritional alternatives to antibiotics remains highly variable across farms and production systems, suggesting that host- and microbiota-related factors may critically modulate their biological effects [6].

Tannins are broadly classified into hydrolysable tannins (HT) and condensed tannins (CT). HT include gallotannins and ellagitannins, which are esters of gallic acid or hexahydroxydiphenoyl/ellagic acid with a polyol, typically glucose. Chestnut wood extracts are particularly rich in ellagitannins, with castalagin and vescalagin among their major constituents. By contrast, CT, also referred to as proanthocyanidins, are non-hydrolysable oligomers and polymers of flavan-3-ols linked by C–C bonds; quebracho (*Schinopsis* spp.) heartwood is a well-known source of CT-rich extracts [3, 7, 8]. The structural differences between HT and CT are nutritionally relevant, as they influence chemical reactivity, microbial accessibility, and downstream metabolic fate in the gut.

Evidence from pig studies indicates that both HT and CT can exert beneficial effects on intestinal health, although responses depend on dose, formulation, and production context [3, 4]. In an ETEC F4 challenge model, dietary chestnut HT reduced fecal scores, diarrhea incidence, and diarrhea duration without compromising feed intake (FI) or average daily gain (ADG) [9]. In commercial or farm-like settings, quebracho-derived CT have also been reported to reduce diarrhea and improve intestinal morphology, including villus and crypt traits, again with limited or no negative effects on growth performance [10, 11]. Likewise, supplementation with a chestnut–quebracho blend changed the fecal microbiota of weaned piglets, favoring butyrate-associated taxa and increasing fecal butyrate concentration [12]. Taken together, these studies suggest that tannins can improve gut resilience during weaning, although some variability among studies remains. This variability may be partly explained by differences in the baseline capacity of the gut microbiota to metabolize tannins. Indeed, an important but still underexplored aspect is that the biological effects of tannins are mediated not only by the parent compounds but also by their microbial catabolites. Ellagitannin-rich HT are converted by the gut microbiota into urolithins (Uros), including urolithin A (Uro-A), urolithin B (Uro-B), urolithin C (Uro-C), and isourolithin A (IsoUro-A). In parallel, condensed tannins are extensively metabolized into other phenolic metabolites, such as valerolactones, valeric acids, and smaller phenolic acids [13–15].

Among tannin-derived microbial metabolites, urolithins represent one of the best-characterized examples of microbiota-dependent polyphenol biotransformation. These metabolites are increasingly recognized as relevant bioactive mediators of host responses. In rodents, Uro‑A has been shown to improve intestinal barrier function, including effects on tight-junction and mucus-related programs, and to attenuate colitis severity [16–18]. Likewise, dietary proanthocyanidins and their catabolites have been linked to improved gut immune homeostasis, lower inflammation, and better barrier integrity [19]. Thus, the health effects of dietary tannins may depend heavily on the ability of the resident gut microbiota to convert the ingested polyphenols into active downstream molecules [20].

This microbiota dependence is well illustrated by the concept of urolithin metabotypes (UM). In humans, individuals can be classified as UM-A, UM-B, or UM-0 according to their capacity to convert ellagic acid and ellagitannins into different Uro profiles [21, 22]. The metabotypes are linked to differences in gut microbial configuration and can influence the physiological response to ellagitannin-rich foods [14, 23, 24]. By analogy, farm- or individual-level differences in microbial tannin biotransformation capacity may contribute to the heterogeneous responses observed in tannin-supplemented piglets under commercial conditions.

Based on these considerations, we hypothesized that the biological effects of dietary tannin supplementation in weaned piglets are modulated by their gut microbiota. Therefore, the present study evaluated the effects of a blend of chestnut-derived hydrolysable tannins and quebracho-derived condensed tannins on growth performance, diarrhea occurrence, gut metagenomic structure, and fecal metabolomic profiles in weaned piglets reared under two commercial farm conditions. By integrating growth performance, diarrhea occurrence, shotgun metagenomic profiling of the gut microbiota, and fecal metabolomic data, we aimed to determine whether tannin supplementation enhances post-weaning health in piglets and whether differences in microbial tannin biotransformation, particularly Uro production, account for varying responses to treatment.

## Methods

### Animal ethics

The study protocol was reviewed and approved by the Animal Welfare Committee of the University of Bologna (Project ID 4655; protocol No. 75303/2024, 15 March 2024). The Committee stated that the study was outside the scope of Italian Legislative Decree No. 26/2014 on the use of animals for scientific purposes, pursuant to Article 2.

### Experimental design

At the farrowing facility, 160 piglets were weighed (T0, median age 19.5 [18–21] d, 82 females and 78 castrated males) and individually identified with a numbered ear tag and randomly assigned to 4 experimental groups based on sex, body weight (BW) and litter of origin. The experimental design thus followed a 2 × 2 factorial arrangement, with two farms (weaning units) and two dietary treatments as main factors. At weaning (median age 28 [25–31] d), piglets were moved to the two respective weaning units (W1 and W2; *n* = 80 per unit) and within each weaning unit were divided into a control group (CTR) receiving a standard diet, and a treated group (TRT) receiving the same diet supplemented with 2 g/kg of a chestnut and quebracho extract blend (Silvafeed^®^Nutri P, Silvateam, Italy). In both weaning units, pigs were housed in a single pen per group on fully slatted plastic floors (W1) or partially slatted concrete floors (W2). Environmental conditions were automatically controlled to maintain appropriate temperature; ventilation was natural/forced in both weaning units. Feed (Additional file 1) and water were provided ad libitum through automatic feeders and nipple drinkers. Environmental enrichment was minimal, consisting of iron chains combined with wooden blocks. Space allowance was approximately 0.35 m^2^ per pig, in accordance with the requirements of European Directive 2008/120/EC. Both weaning farms implemented an all-in/all-out management system.

The functional ingredient consisted of a standardized combination of tannin-rich phytocomplexes obtained from quebracho (*Schinopsis lorentzii* Engl.) wood and chestnut (*Castanea sativa* Mill.) extracts (Silvateam S.p.A.), produced via food-grade hot water extraction. The supplement provided 2.98 mmol of gallic acid equivalents per gram (GEAC, Folin–Ciocalteu method [25]) and a minimum tannin content of 65%, as verified by OIV analysis. The quebracho fraction is mainly composed of condensed tannins, such as profisetinidins and fisetin [26], whereas the chestnut fraction predominantly contains ellagitannins, including digalloyl-glucose, glucose, and gallic acid [27].

### Sampling

Pigs were then weighed at d 35 (T1), d 49 (T2) and d 76 (T3). At each time point, fecal samples were collected from all pigs in a sterile tube, immediately frozen in liquid nitrogen, and stored at −80 °C. In addition, fecal consistency was scored using a 5-point scale in which higher scores indicate looser feces (1 = firm, normal feces; 5 = watery, liquid feces). Diarrhea was recorded when the fecal score was 3.5 or higher (i.e., ≥ 3.5) [28]. Overall, the study comprised four experimental groups defined by the 2 × 2 arrangement (CTR_W1, TRT_W1, CTR_W2, TRT_W2; *n* = 40 per group at enrolment). Fecal samples were collected from every animal at each post-weaning time point (T1, T2, T3). Four of the 160 piglets (one from the control and three from the tannin-supplemented group, two in each farm) died or were removed for health reasons during the post-weaning period and were excluded from all analyses; fecal samples were therefore available for 156 animals. The samples were allocated to the analyses as follows: shotgun metagenomics on samples from all 156 animals at each time point; untargeted metabolomics on 200 fecal samples (100 per time point at T1 and T2), a balanced subset of control and treated pigs from both farms selected by proximity to the group-mean average daily gain; and targeted urolithin quantification at T1 and T2 on the tannin-treated pigs (77 per time point; 154 samples) plus six control pigs (three per farm; 12 samples), giving 166 samples in total.

### DNA extraction and shotgun metagenome sequencing

Fecal samples were used to extract the total bacterial DNA according to the manufacturer's instructions using the standard homogenized protocol for fecal samples by the FastPrep-24^TM^ instrument (MP Biomedicals, Santa Ana, CA, USA). DNA was quantified using a QIAxpert microfluidic spectrophotometer (QIAGEN) together with a fluorimetric assay; DNA integrity was not further assessed. Sequencing libraries were prepared with the Illumina DNA Prep (M) Tagmentation kit (Illumina, San Diego, CA, USA; Cat. No. 20060059) according to the manufacturer's instructions. All samples were processed identically; the only amplification was the standard indexing PCR intrinsic to the tagmentation workflow, applied uniformly to every library, so no subset of samples underwent selective or additional amplification. Libraries were purified, pooled at an equimolar concentration, and sequenced on an Illumina NovaSeq 6000Dx using a 2 × 150 bp paired-end protocol to obtain > 4 Gb per sample.

### Untargeted metabolomic analysis

Frozen fecal samples were shipped on dry ice to Metabolon, Inc. (Morrisville, NC, USA) for untargeted UPLC-MS/MS analysis. Upon receipt, samples were inventoried and stored at −80 °C. Samples were then processed using the automated MicroLab STAR^®^ system (Hamilton Company). Several recovery standards were added for quality control (QC) purposes. Proteins were precipitated with methanol, and the resulting extract was divided into fractions for analysis via reverse-phase (RP) and hydrophilic interaction liquid chromatography (HILIC) coupled with UPLC-MS/MS in both positive and negative ionization modes. Organic solvents were removed, and extracts were stored overnight under nitrogen prior to analysis. Control samples, including pooled matrix samples, process blanks, and QC standards, were analyzed alongside experimental samples to monitor instrument performance and process variability. Instrument variability was assessed using the median relative standard deviation (RSD) of internal standards, and overall process variability was determined by the median RSD of endogenous metabolites in pooled samples.

Metabolites were analyzed using UPLC coupled with high-resolution mass spectrometry. Four separate methods were employed, utilizing both positive and negative ionization modes. Samples were gradient eluted from different chromatographic columns, including C18 and HILIC, and raw data were collected covering a mass range of 70–1,000 *m/z*. Data extraction, peak identification, and QC were performed using Metabolon’s proprietary software. Compounds were identified based on retention index, accurate mass match to the library (*m/z*), and MS/MS spectral comparison against an extensive reference library. For multi-day studies, data normalization was performed to correct for instrument variation by registering the medians to one and applying block corrections. Metabolite semi-quantification was based on relative area-under-the-curve integration, after data normalization.

### Urolithins quantification

Approximately 100 mg of lyophilized feces from TRT piglets reared in farms W1 and W2 at T1 and T2 were extracted with 1 mL of a methanol/water mixture (MeOH/H_2_O, 80:20, v/v) acidified with 0.1% hydrochloric acid. In addition, fecal samples from six CTR piglets (three per farm), collected at T1 and T2 (12 samples in total), were also analyzed to ensure no Uro detection. Samples were vortex-mixed for 2 min, sonicated in an ultrasonic bath for 5 min, and subsequently centrifuged at 14,000 × *g* for 10 min at room temperature.

The resulting supernatants were collected and filtered through a 0.22-µm PVDF membrane prior to chromatographic analysis by UPLC-ESI-QTOF-MS.

Ellagic acid and its derived metabolites were analyzed using an optimized method for the quantification of Uros and ellagic acid in biological matrices, as previously described by García-Villalba et al. [29]. Analyses were performed using an Agilent 1290 Infinity LC system coupled to a 6550 iFunnel Accurate-Mass QTOF mass spectrometer (Agilent Technologies, Waldbronn, Germany) equipped with a dual electrospray ionization interface (ESI-Jet Stream Technology), allowing continuous mass axis calibration through the simultaneous infusion of a reference mass solution. Chromatographic separation was achieved on a Poroshell 120 EC-C18 column using water containing 0.1% formic acid (solvent A) and acetonitrile containing 0.1% formic acid (solvent B) under gradient elution conditions.

Data acquisition and processing were carried out using MassHunter Qualitative Analysis software (version B.10, Agilent Technologies). A targeted screening strategy was applied to all samples for the identification of ellagic acid and its derived metabolites (Uros). Quantification of urolithins and EA was performed by interpolation from their corresponding external calibration curves.

### Statistical analysis on performance and health parameters

All statistical analyses were performed using R version 4.4.0 (R Foundation for Statistical Computing, Vienna, Austria). The following packages were used: lme4 (v1.1-37), car (v3.1-3), nortest (v1.0-4), lsmeans (v2.30-2), multcompView (v0.1-10), multcomp (v1.4-29), nlme (v3.1-164), jmv (v2.7.7) and mixOmics (v6.28.0).

Body weight (BW) at each time point (T0, T1, T2, T3) and ADG over the different post-weaning intervals were analyzed using linear mixed-effects models fitted with the lmer function of the lme4 package. The general model was:

$${Y}_{ijklm}=\mu +{F}_{i}+{D}_{j}+{S}_{k}+{\left(F\times D\right)}_{ij}+{u}_{m\left(l\right)}+\varepsilon ijklm$$

where *Y*_*ijklm*_ is the response variable (BW or ADG) observed on the *m*-th pig within the *l*-th random unit; *μ* is the overall mean; *F*_*i*_ is the fixed effect of the *i*-th farm (W1, W2); *D*_*j*_ is the fixed effect of the *j*-th dietary treatment (CTR, TRT); *S*_*k*_ is the fixed effect of the *k*-th sex (female, castrated male); *(F × D)*_*ij*_ is the farm × dietary treatment interaction; *u*_*m(l)*_ is the random effect of the *l*-th sow (litter of origin); because each farm × diet group was housed in a single pen, pen was confounded with the farm × diet combination and was not included as a random effect, assumed *u*_*m*__(__*l*__)_ ~ N(0, σ^2^u); and *εijklm* is the residual error, assumed *εijklm* ~ N(0, σ^2^ε). Model assumptions were evaluated by visual inspection of residual plots (normal Q–Q plots and residual versus fitted plots) and by Shapiro–Wilk tests for normality. Fixed effects were tested using Type III analysis of variance. When significant main effects or interactions were detected, least-squares means were calculated using the *lsmeans* package, and pairwise comparisons were adjusted according to Tukey’s method. Results are presented as least-squares means, and statistical significance was declared at *P* < 0.050. Diarrhea occurrence at T1 and T2 was analyzed using generalized linear mixed models with a binomial distribution and logit link function, fitted with the glmer function of the lme4 package. The model was:

$$logit\left({\pi}_{ijkl}\right)=\mu +{F}_{i}+{D}_{j}+{S}_{k}+{\left(F\times D\right)}_{ij}+{u}_{l}$$

where *π*_*ijkl*_ is the probability of a diarrhea event for the pig in the *i*-th farm, *j*-th dietary treatment, *k*-th sex, and *l*-th sow (litter of origin); *F*_*i*_, *D*_*j*_, *S*_*k*_ and *(F × D)*_*ij*_ are defined as above; and *u*_*l*_ is the random effect of the *l*-th sow (litter of origin), assumed *u*_*l*_ ~ N(0, σ^2^u). When no diarrhea cases were recorded in one farm, the analysis was performed within the remaining farm only. Least-squares means were obtained on the response (probability) scale, and pairwise comparisons were adjusted using Tukey’s method. Type III analysis of variance was used to test fixed effects. Statistical significance was set at *P* < 0.050.

### Bioinformatic analysis on the gut microbiome

Species-level characterization of shotgun metagenomic data was conducted as follows. Read quality was assessed with FastQC (v0.11.9), reads were quality-trimmed and filtered with fastp (v0.23.4), and host reads were removed with KneadData (v0.12.0) using the Sscrofa11.1 genome (GCF_000003025.6) as reference, following the standard operating procedures of the HMP Consortium [30]. The retained reads were taxonomically characterized at species level by MetaPhlAn4 [31] using the mpa_vOct22_CHOCOPhlanSGB database. Metagenomes were functionally profiled using HUMAnN3 [32] with the UniRef90 database (uniref90_v2019) to quantify the abundance of genes and pathways. Reads were aligned to sample-specific pangenomes, i.e., all gene families in any microorganism detected in a given sample, using Bowtie2 and the UniRef90, MinPath and KEGG databases [33–36]. Hits were counted per KEGG pathway and KO genes and normalized for length, alignment quality score and sequencing depth.

For the taxonomic composition, alpha diversity measures, including Shannon and Inverse Simpson indices, were calculated for each time point. The effect of treatment, farm, and their interaction was assessed using a linear mixed-effects model, with litter of origin included as a random effect. Beta diversity was calculated at the species level for each time point using the Bray–Curtis distance matrix and analyzed with a PERMANOVA (*adonis.test* function), incorporating treatment, farm and their interaction, as fixed effects. When a significant treatment × farm interaction occurred, PERMANOVA contrasts between CTR and TRT within each farm were performed using *pairwise.adonis* function included in pairwiseAdonis_0.4.1 package in R. Results were visualized using Principal Coordinate Analysis (PCoA). Microbial markers for each time point were identified using LEfSe, retaining only markers with a linear discriminant analysis (LDA) score > 3.5 and an adjusted *P* < 0.050 [37]. For the functional profile, alpha diversity and beta diversity were measured and analyzed using the same structure as for the taxonomic composition, while KOs associated with each treatment, for each time point, were identified using DESeq2, retaining pathways with log fold change (logFC) > 2 or < −2 and an adjusted *P* < 0.050. Functional enrichment was conducted to identify over-represented biological pathways and the analysis was specifically restricted to Metabolism (ko01), Genetic Information Processing (ko02), Environmental Information Processing (ko03), and Cellular Processes (ko04) to ensure biological relevance to the porcine gut microbial environment. The Gene Set Enrichment Analysis (GSEA) was executed through the *clusterProfiler* package in R, with all KOs ranked by their log2_FC values derived from the DESeq2 models. The enrichment score was normalized (NES) to account for the size of each gene set, and pathways were declared significantly enriched if the BH-adjusted *P* was < 0.050.

### Statistical analysis on fecal metabolomics

A Sparse Partial Least Square Discriminant Analysis (sPLS-DA) was used to investigate differences between the metabolomic profiles of the 4 experimental groups within each time point. The optimal number of metabolites to use was determined during the tuning process, which was run using fivefold cross validation and 50 repeats. Metabolites contributing with non-null coefficients to discriminant dimensions were selected and validated by applying a permutation test coupled with a M-fold cross-validation. Most discriminant loading features in the sPLS-DA model were computed and plotted using the mixOmics “plot loading” function. All analyses have been performed in R v. 4.4.0 using the “mixOmics” package for the sPLS-DA analysis.

### Correlation between urolithins and metagenomic data

To identify taxa or functional genes associated to Uro production we stratified pigs according to their different capacities to metabolize ellagic acid derivatives into Uros, i.e. urolithin metabotypes (UM-A, -B or -0), they were assigned to UM-B when either Uro-B or IsoUro-A was detected, to UM-A when Uro-A was detected in the absence of Uro-B and IsoUro-A, and to UM-0 when none of these metabolites were detected [38]. We then restricted the analysis to piglets identified as UM-B and to T2, when Uro production was more pronounced. Within this subset, concentrations of ellagic acid, IsoUro-A, Uro-A, Uro-B, and Uro-C were compared between W1 and W2 at each time point using the Wilcoxon rank-sum test. Moreover, we performed a weighted gene co-expression network analysis (WGCNA) in R 4.4 using the *WGCNA* package (v1.73) on species and KOs abundance matrices. Prior to network construction, an appropriate soft-thresholding power was determined to approximate scale-free network topology. Candidate powers were evaluated using the *pickSoftThreshold* function across a range of values (1–10, 12, 14, 16, 18, and 20). The selection was guided by inspection of the scale-free topology fit index and mean connectivity, identifying the lowest power that achieved a satisfactory approximation of scale-free topology while maintaining adequate network connectivity. Signed co-expression networks were then constructed separately for species and KO profiles using the *blockwiseModules* function. The following parameters were applied: networkType = "signed", minModuleSize = 20, mergeCutHeight = 0.25, deepSplit = 2, pamRespectsDendro = FALSE, maxBlockSize = 4,000, and reassignThreshold = 0. Module eigengenes were calculated for each detected module and used as representative profiles for downstream analyses. To investigate associations between microbial network structure and metabolite profiles, module eigengenes from the species and KO networks were combined with log-transformed Uro metabolite abundances. The metabolite matrix included ellagic acid, IsoUro-A, Uro-A, Uro-B, and Uro-C. Pearson correlation coefficients were calculated using pairwise complete observations. Correlation matrices were visualized with the *corrplot* package (v0.95). Modules of interest from both the species and KOs WGCNA networks were identified based on their Pearson correlations with urolithin-related metabolites (*r* ≥ 0.4). For the selected modules, intramodular network structure was extracted by exporting topological overlap matrix (TOM)-based edge lists from the WGCNA analysis. Network visualization and centrality analyses were performed in Python 3.14.3 using the packages pandas (v3.0.0), networkx (v3.6.1), matplotlib (v3.10.8), scikit-learn (v1.8.0), and adjustText (v1.3.0). Self-loops were removed and edges were filtered using module-specific TOM similarity thresholds to retain the strongest connections within each module. Undirected weighted graphs were then constructed using networkx.from_pandas_edgelist, with TOM similarity stored as the edge weight attribute. To identify highly connected nodes, four node-level centrality measures were computed: degree centrality, betweenness centrality, closeness centrality, and eigenvector centrality. If eigenvector centrality could not be reliably estimated for a network, nodes were assigned a value of zero for this metric. Centrality scores were normalized using min–max scaling (sklearn.preprocessing.MinMaxScaler), and a combined centrality score was calculated as the arithmetic mean of the four normalized metrics. Nodes with the highest combined scores were considered candidate hub taxa or functional genes. Network layouts were generated using the Kamada–Kawai force-directed algorithm (nx.kamada_kawai_layout). In the graphical representations, top-ranked hub nodes were highlighted, and node labels were adjusted using the adjustText library to minimize overlap.

## Results

### Growth performance and health

As shown in Table 1, farm (W1 vs. W2) significantly affected growth performance parameters. Body weight differed between farms at T2 and T3 (*P* < 0.01), with pigs reared in W1 being heavier than those in W2 at both time points. Similarly, ADG was consistently higher in W1 compared with W2 during T0–T2, T0–T3, T1–T2, T1–T3, and T2–T3 (*P* < 0.01 for all comparisons). A farm effect on body weight was also detected at T1 (*P* = 0.001), with pigs in W2 being heavier than those in W1. A significant farm effect was also observed for diarrhea occurrence at T1 (*P* < 0.01), with pigs in W1 exhibiting a higher prevalence of diarrhea compared with pigs in W2 during the first week post-weaning. At T2, dietary treatment (CTR vs. TRT) significantly affected diarrhea occurrence (*P* = 0.032); pigs receiving the TRT diet showed a lower prevalence of diarrhea compared with CTR pigs in W2, whereas no diarrhea was recorded in W1 at this time point. No diarrhea was recorded at T3 in either farm. A higher average daily gain was observed in tannin-supplemented pigs during the T1–T2 interval; no other effect of dietary treatment on growth was detected (*P* > 0.050).

**Table 1:** Effects of farm, dietary treatment, sex and the interaction between rearing farm and dietary treatment on pigs' productive performance and occurrence of diarrhea during post-weaning period at each time points

Item	W1^1^		W2		SEM	*P*-values^3^			
	CTR^2^	TRT	CTR	TRT		F	D	S	F × D
Body weight, kg									
T0^4^	6.53	6.53	6.55	6.53	0.133	0.929	0.971	0.752	0.917
T1	8.47	8.32	8.94	8.96	0.214	0.001	0.691	0.221	0.585
T2	14.0	14.1	11.3	12.0	0.327	< 0.001	0.153	0.151	0.205
T3	34.5	35.2	28.0	28.2	0.773	< 0.001	0.472	0.540	0.732
Average daily gain, g/d									
T0–T1	137	125	158	160	9.89	0.001	0.568	0.026	0.422
T0–T2	266	268	162	188	9.33	< 0.001	0.127	0.050	0.163
T0–T3	500	511	376	380	12.67	< 0.001	0.454	0.447	0.727
T1–T2	397	411	166	219	15.10	< 0.001	0.026	0.347	0.201
T1–T3	621	640	453	458	15.21	< 0.001	0.343	0.708	0.571
T2–T3	732	754	596	577	19.14	< 0.001	0.917	0.967	0.192
Diarrhea occurrence^5^, %									
T1	23.1	39.5	10.3	15.8	8.01	0.010	0.155	0.412	0.758
T2^6^	-	-	20.0	2.6	6.15	-	0.032	0.163	-

^1^W1 = Weaning Unit 1; W2 = Weaning Unit 2

^2^CTR = pigs fed the basal diet; TRT = pigs fed the basal diet plus 2 g/kg chestnut–quebracho tannin blend

^3^F = farm effect; D = dietary treatment effect; S = sex effect; F × D = farm × dietary treatment interaction. No diarrhea was recorded at T3 in either farm

^4^T0 = farrowing unit; T1 = d 35; T2 = d 49; T3 = d 76

^5^Diarrhea was declared when fecal score was > 3.5

^6^ Hyphen indicates no diarrhea cases recorded

Given the higher prevalence of diarrhea observed in W1 at T1, antibiotic treatments were administered exclusively in W1 at T2 in both dietary groups (CTR_W1 and TRT_W1). Lincomycin (10 mg/kg BW/d) and spectinomycin (20 mg/kg BW/d) were administered via drinking water for 5 consecutive days, followed by amoxicillin (15–20 mg/kg body weight/d) for an additional 5 d. No antibiotic treatments were recorded at T0 or T1 in either farm, nor in W2 at any time point.

### Metagenomic profile

Illumina shotgun sequencing of the fecal samples yielded, on average, 48.3 ± 6.8 million paired-end reads per sample; after quality control and host-read removal, 40.0 ± 5.5 million reads per sample were retained (mean mapping rate 2.85% ± 0.50%). Three samples that did not yield a sufficient number of reads were excluded from the analyses (two from TRT_W1, at T1 and T2, and one from TRT_W2, at T3), leaving 465 samples (155 per time point). Taxonomic profiling with MetaPhlAn4 identified 1,268 bacterial species, while functional annotation with HUMAnN3 identified 44,498 KEGG Orthologs (KOs).

#### Taxonomic level

**Fig. 1:**
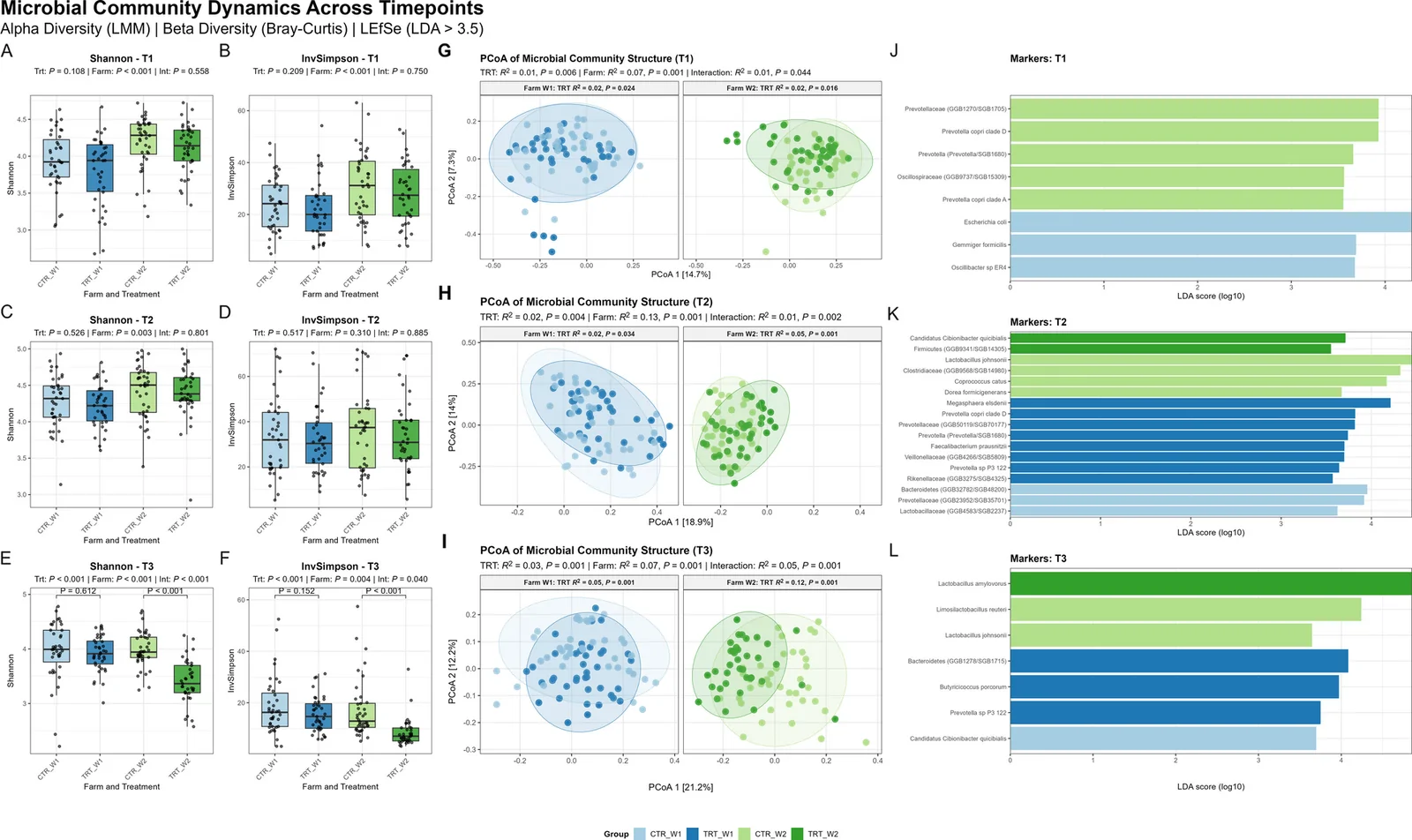
Effects of experimental treatment and farming conditions on species-level gut microbiota composition across time points. **A**–**F** Alpha diversity (Shannon and inverse Simpson indices) calculated from MetaPhlAn species-level profiles and analyzed using linear mixed-effects models including treatment (CTR vs. TRT), farm (W1 vs. W2), and their interaction as fixed effects, with sow as a random effect. **G**–**I** Beta diversity visualized by principal coordinate analysis based on Bray–Curtis dissimilarity. Permutational multivariate analysis of variance was used to assess the effects of treatment, farm, and their interaction, with additional pairwise comparisons performed within each farm. **J–L** Differentially abundant species identified using linear discriminant analysis effect size, retaining taxa with linear discriminant analysis score > 3.5 and adjusted *P* < 0.01. CTR = pigs fed the basal diet; TRT = pigs fed the basal diet plus 2 g/kg chestnut–quebracho tannin blend; W1 and W2 = commercial weaning units

Alpha diversity analysis revealed that at T1 (Fig. 1A and B), both Shannon and Inverse Simpson indices were significantly affected by farm (*P* < 0.001), with lower values observed in W1 compared to W2, and no significant effects of treatment or interaction. At T2 (Fig. 1C and D), Shannon diversity was affected by farm (*P* = 0.003), again with lower values in W1 compared to W2, while no significant effects were observed for treatment, interaction, or the Inverse Simpson index. At T3 (Fig. 1E and F), significant effects of treatment, farm, and their interaction were detected for both diversity indices. For the Shannon index, significant effects were observed for treatment (*P* < 0.001), farm (*P* < 0.001), and the treatment × farm interaction (*P* < 0.001). Similarly, the Inverse Simpson index was significantly affected by treatment (*P* < 0.001), farm (*P* = 0.004), and their interaction (*P* = 0.040). Within-farm contrasts indicated a significant reduction in both Shannon (*P* < 0.01) and Inverse Simpson (*P* < 0.01) indices in W2, whereas no significant effect was observed in W1.

PCoA plots of species-level bacterial beta diversity at T1, T2, and T3 are presented in Fig. 1G–I. PERMANOVA analysis at T1 indicated that bacterial composition was significantly affected by treatment (*P* < 0.01, *R*^2^ = 0.01), farm (*P* = 0.001, *R*^2^ = 0.07), and their interaction (*P* = 0.048, *R*^2^ = 0.01). Pairwise contrasts performed within each farm showed that the treatment effect was consistent in both W1 (*P* < 0.01, *R*^2^ = 0.01) and W2 (*P* = 0.041, *R*^2^ = 0.02). At T2, bacterial composition was significantly influenced by treatment (*P* < 0.01, *R*^2^ = 0.02), farm (*P* = 0.001, *R*^2^ = 0.13), and their interaction (*P* < 0.01, *R*^2^ = 0.01). Pairwise contrasts within each farm confirmed a consistent treatment effect in both W1 (*P* < 0.01, *R*^2^ = 0.02) and W2 (*P* < 0.01, *R*^2^ = 0.01). At T3, bacterial composition remained significantly affected by treatment (*P* = 0.001, *R*^2^ = 0.03), farm (*P* = 0.001, *R*^2^ = 0.07), and their interaction (*P* = 0.001, *R*^2^ = 0.05). Pairwise contrasts within each farm again demonstrated a consistent treatment effect in W1 (*P* < 0.01, *R*^2^ = 0.02) and W2 (*P* < 0.01, *R*^2^ = 0.01).

LEfSe identified differentially abundant taxa at each time point (Fig. 1J–L); for the main discriminant species, mean relative abundances (± SD) in the enriched versus the contrasting group are reported below. At T1, control pigs were enriched in *Escherichia coli* (LDA = 4.29; 1.7% ± 2.4% vs. 0.6% ± 1.5% in tannin-fed pigs) in W1 and in *Prevotella copri* clades (1.7% ± 1.9% vs. 0.8% ± 0.9%) in W2. At T2, tannin-fed pigs in W1 were enriched in fiber-fermenting and short-chain fatty acid–producing taxa, most notably *Megasphaera elsdenii* (LDA = 4.21; 2.4% ± 1.7% vs. 2.0% ± 1.8% in controls) and *Faecalibacterium prausnitzii* (1.2% ± 0.9% vs. 1.0% ± 0.9%), whereas control pigs in W2 were enriched in *Lactobacillus johnsonii* (3.2% ± 2.3% vs. 2.7% ± 2.1%) and *Coprococcus catus* (1.5% ± 1.2% vs. 0.8% ± 0.7%); tannin-fed pigs in W2 were enriched in *Candidatus Cibionibacter quicibialis* (1.0% ± 0.7% vs. 0.5% ± 0.3% in controls). At T3, tannin-fed pigs were enriched in *Butyricicoccus porcorum* (W1; 1.4% ± 1.3% vs. 0.4% ± 0.5% in controls) and *Lactobacillus amylovorus* (W2; LDA = 4.85; 24.4% ± 6.5% vs. 14.9% ± 5.6%), while control pigs were enriched in *Limosilactobacillus reuteri* (W2; 2.9% ± 1.3% vs. 2.2% ± 1.0% in tannin-fed pigs). The complete set of discriminant taxa with their LDA scores is shown in Fig. 1J–L, and the fully annotated list is provided in Additional file 2.

#### Functional level

**Fig. 2:**
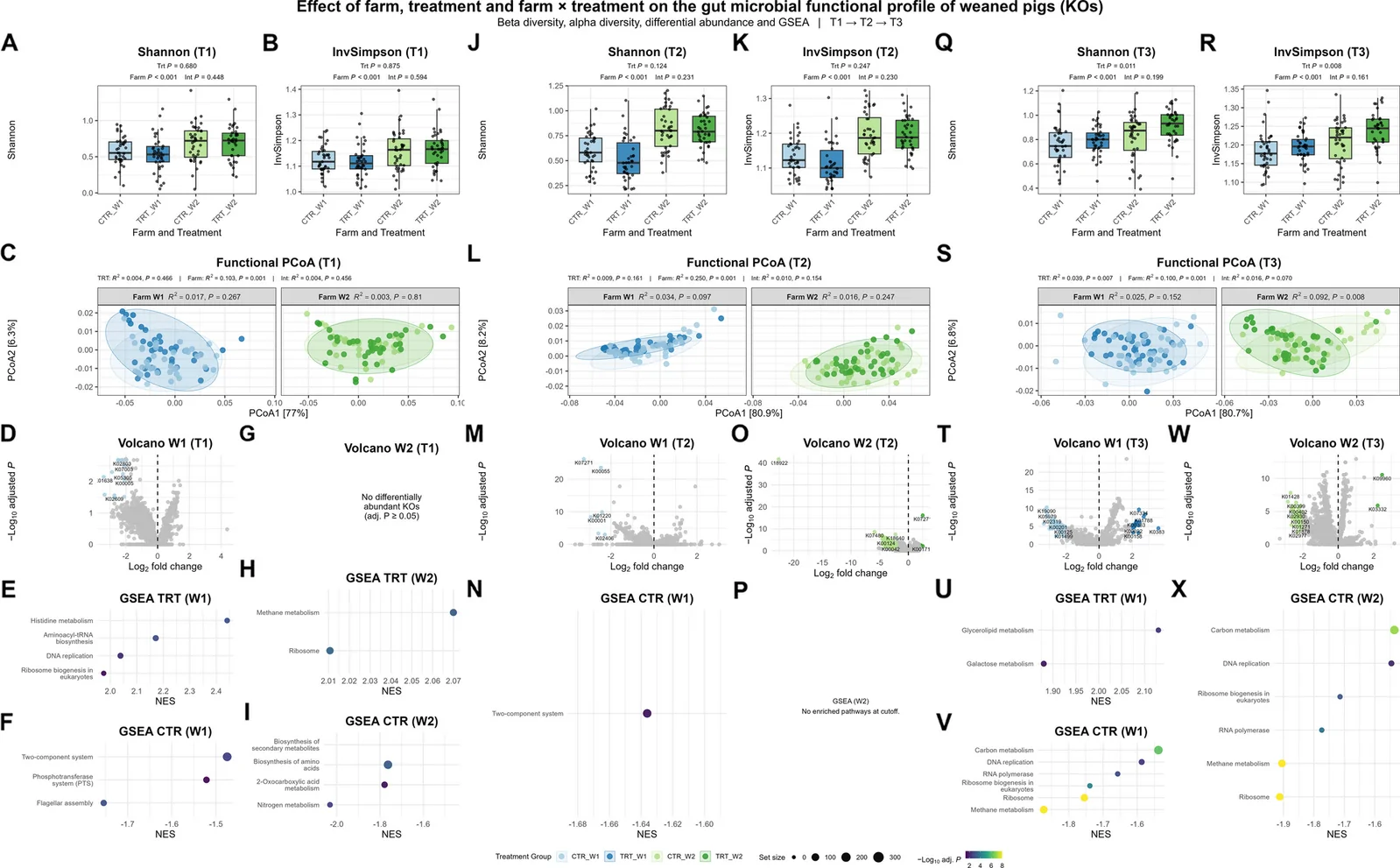
Effects of treatment, farm, and treatment × farm interaction on piglet gut microbial functional profile. **A**, **B**, **J**, **K**, **Q**, **R** Alpha diversity of KEGG Ortholog profiles shown as boxplots with jittered points. **C**, **L**, **S** Beta diversity of KEGG Ortholog profiles visualized by principal coordinate analysis based on Bray–Curtis dissimilarity; permutational multivariate analysis of variance was used to test the effects of treatment, farm, and their interaction, and pairwise comparisons were performed within each farm. **D**, **G**, **M**, **O**, **T**, **W** Differentially abundant KEGG Orthologs between treatment groups within each farm were identified by DESeq2 and visualized as volcano plots, with significance declared at false discovery rate-adjusted *P* < 0.050 and |log_2_ fold change|≥ 1. **E**, **F**, **H**, **I**, **N**, **P**, **U**, **V**, **X **KEGG pathway enrichment was assessed by gene set enrichment analysis using ranked DESeq2 log_2_ fold changes; dotplots show normalized enrichment score, pathway set size, and adjusted *P* values. CTR = pigs fed the basal diet; TRT = pigs fed the basal diet plus 2 g/kg chestnut–quebracho tannin blend; W1 and W2 = commercial weaning units

Alpha diversity analysis revealed a consistent farm effect across all sampling times. At T1, both Shannon (Fig. 2A) and InvSimpson (Fig. 2B) indices were lower in W1 than in W2 (*P* < 0.001), whereas no effects of dietary treatment or sex were detected. A similar pattern was observed at T2 for both diversity indices (Fig. 2J and K; *P* < 0.001). At T3, alpha diversity remained lower in W1 compared with W2 and was additionally influenced by dietary treatment, with lower Shannon (*P* = 0.010) and InvSimpson (*P* = 0.008) values in CTR compared with TRT pigs (Fig. 2Q and R). The PERMANOVA analysis of beta diversity on KO profiles indicated that the overall functional profile was affected by farm at T1 (*P* = 0.001, *R*^2^ = 0.10) while no effect for the other factors was evidenced at this time point (Fig. 2C). At T2 the beta diversity was affected by the farm (*P* = 0.001, *R*^2^ = 0.10) (Fig. 2L) and no effect for the other factors was observed. At T3, the beta diversity was affected by the farm (*P* = 0.001, *R*^2^ = 0.10) and treatment (*P* = 0.007, *R*^2^ = 0.04) and tended to be affected by their interaction (*P* = 0.070, *R*^2^ = 0.02) (Fig. 2T).

Differential abundance analysis of KEGG Orthologs (KOs) between TRT and CTR was performed separately within each farm (W1 and W2) at each time point using DESeq2, and functional enrichment was assessed by GSEA on ranked log_2_ fold-changes.

At T1, 79 KOs differed significantly in W1 (all downregulated in TRT), whereas none reached significance in W2. In W1, GSEA indicated TRT enrichment of amino acid metabolism (histidine), aminoacyl-tRNA biosynthesis and DNA replication, and CTR enrichment of flagellar assembly, phosphotransferase and two-component systems (Fig. 2E and F). In W2, despite the absence of individually significant KOs, GSEA indicated TRT enrichment of methane metabolism and ribosome functions and CTR enrichment of nitrogen and amino acid biosynthesis pathways (Fig. 2H and I). The complete list of significant KOs at T1, T2 and T3 is provided in Additional file 3.

At T2, 257 KOs differed in W1 (all downregulated in TRT) and 1,301 in W2 (4 upregulated and 1,297 downregulated in TRT); the only pathway meeting the enrichment threshold was the CTR-enriched two-component system in W1 (Fig. 2N).

At T3, 669 KOs differed in W1 (19 upregulated and 650 downregulated in TRT) and 1,083 in W2 (2 upregulated and 1,081 downregulated). In W1, TRT-enriched pathways were related to carbohydrate metabolism (glycerolipid, galactose, and amino/nucleotide sugar metabolism), whereas CTR-enriched pathways centered on translation and core energy metabolism (ribosome, RNA polymerase, DNA replication, carbon and methane metabolism) (Fig. 2U and V). In W2, CTR-enriched pathways were similar (ribosome, methane and carbon metabolism, RNA polymerase, TCA cycle), with no TRT-enriched pathways (Fig. 2X). Full results are reported in Additional file 3.

### Fecal metabolomics

#### Untargeted metabolomics

To investigate the effect of treatment and farm on fecal metabolomics profile, a sPLS-DA model was constructed at T1 and T2.

**Fig. 3:**
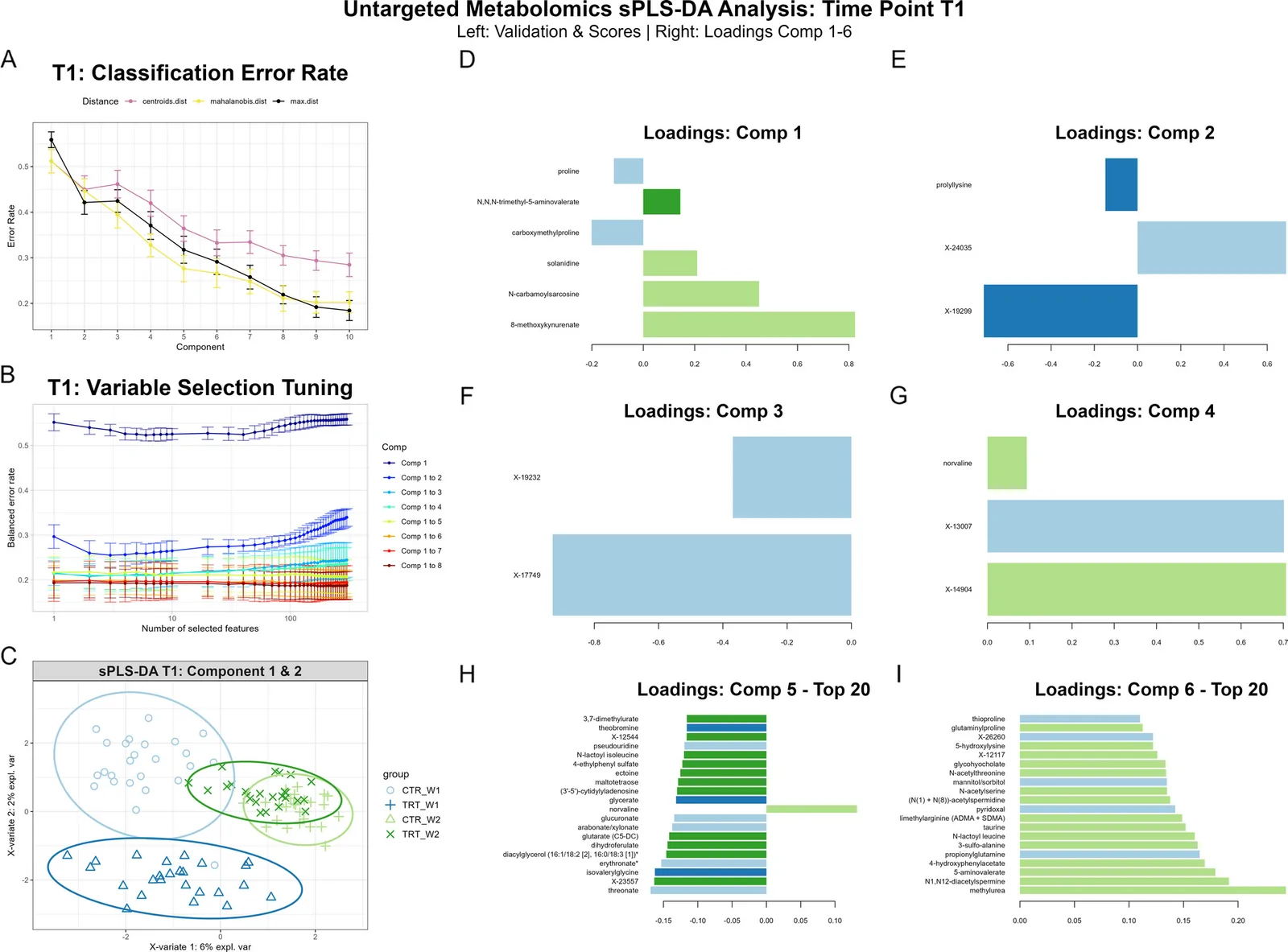
Untargeted metabolomics sPLS-DA analysis at time point T1. Sparse Partial Least Squares Discriminant Analysis (sPLS-DA) was performed to discriminate experimental groups defined by farm (W1 vs. W2) and treatment (CTR vs. TRT). Left panels show model validation and classification performance: **A** balanced error rate (BER) across increasing numbers of components obtained by repeated M-fold cross-validation; **B** tuning results for variable selection (keepX), showing cumulative BER with standard deviation; and (**C**) individual score plot for the optimal model (components 1 and 2). **D**–**I** Right panels show loading plots for components 1 to 6, highlighting the most discriminative metabolites selected by the tuned sPLS-DA model. CTR = pigs fed the basal diet; TRT = pigs fed the basal diet plus 2 g/kg chestnut–quebracho tannin blend; BER = balanced error rate

After cross-validated tuning, the final sPLS-DA model for T1 retained six components, with selection of 6, 3, 2, 3, 270 and 250 variables on components 1–6, respectively. Model performance assessed by repeated M-fold cross-validation yielded a BER of 0.29 ± 0.03 (Fig. 3A–C).

At T1, the most discriminant metabolites of the control groups included 8-methoxykynurenate, N-carbamoylsarcosine and several unidentified features, whereas the tannin groups were characterized by N,N,N-trimethyl-5-aminovalerate, isovalerylglycine and additional features (Fig. 3D–I). The complete loadings are shown in Fig. 3D–I.

**Fig. 4:**
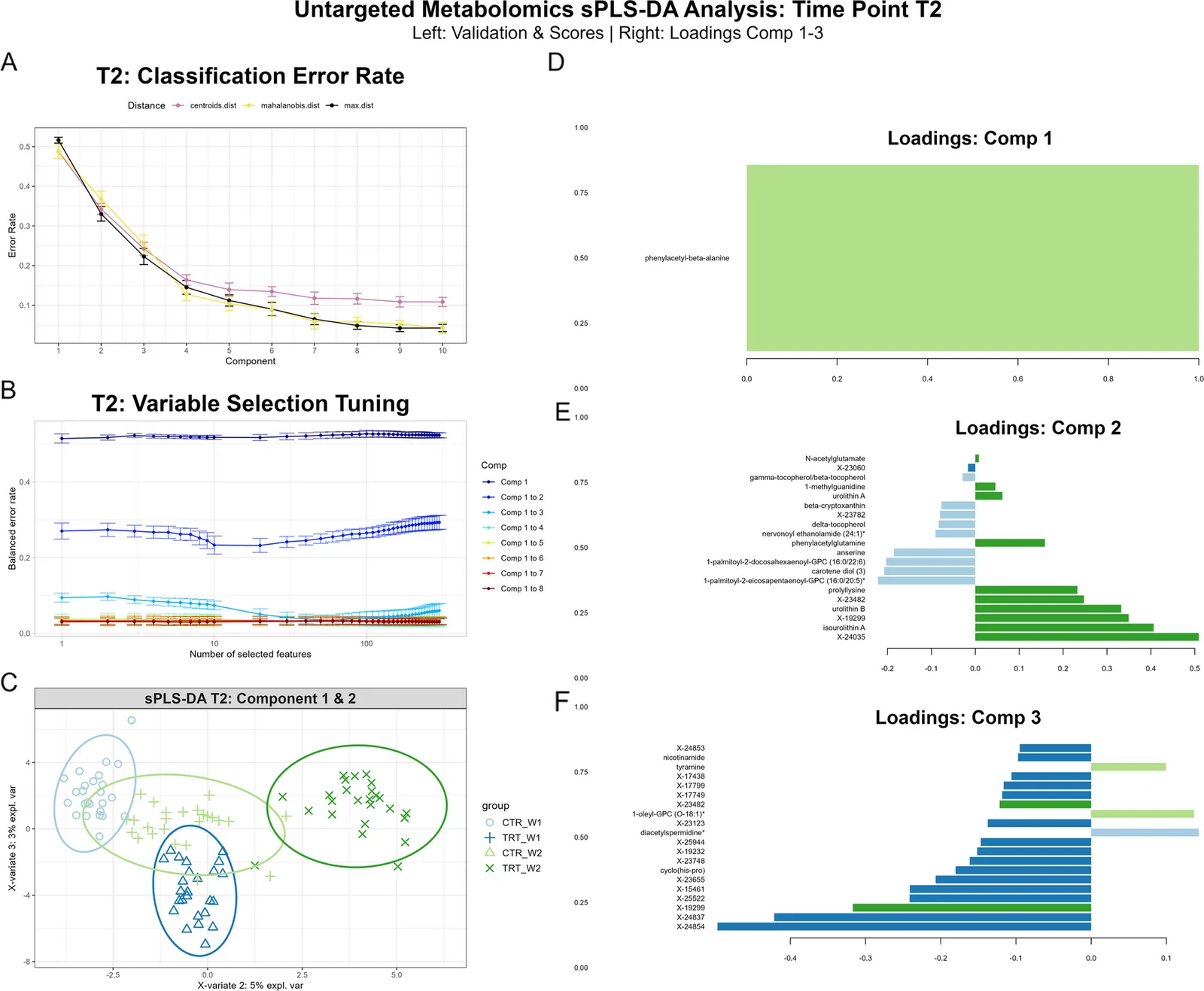
Untargeted metabolomics sPLS-DA analysis at time point T2. Sparse Partial Least Squares Discriminant Analysis (sPLS-DA) was performed to discriminate experimental groups defined by farm (W1 vs. W2) and treatment (CTR vs. TRT). Left panels show model validation and classification performance: **A** balanced error rate (BER) across increasing numbers of components obtained by repeated M-fold cross-validation; **B** tuning results for variable selection (keepX), showing cumulative BER with standard deviation; and **C** individual score plot for the optimal model based on components 1 and 2. **D–F **Right panels show loading plots for components 1 to 3, highlighting the most discriminative metabolites selected by the tuned sPLS-DA model. CTR = pigs fed the basal diet; TRT = pigs fed the basal diet plus 2 g/kg chestnut–quebracho tannin blend; BER = balanced error rate

For T2, the optimized sPLS-DA model retained three components with selection of 1, 20 and 60 variables on components 1–3, respectively. Cross-validated performance showed a BER of 0.22 ± 0.02 (Fig. 4A–C).

At T2, phenylacetyl-β-alanine discriminated the control group, whereas the tannin groups—particularly TRT_W2—were characterized by the ellagitannin-derived urolithins IsoUro-A and Uro-B, together with several unidentified features (Fig. 4D–F). All discriminant features are shown in Fig. 4D–F.

#### Targeted metabolomics and urolithin metabotype (UM) identification

**Fig. 5:**
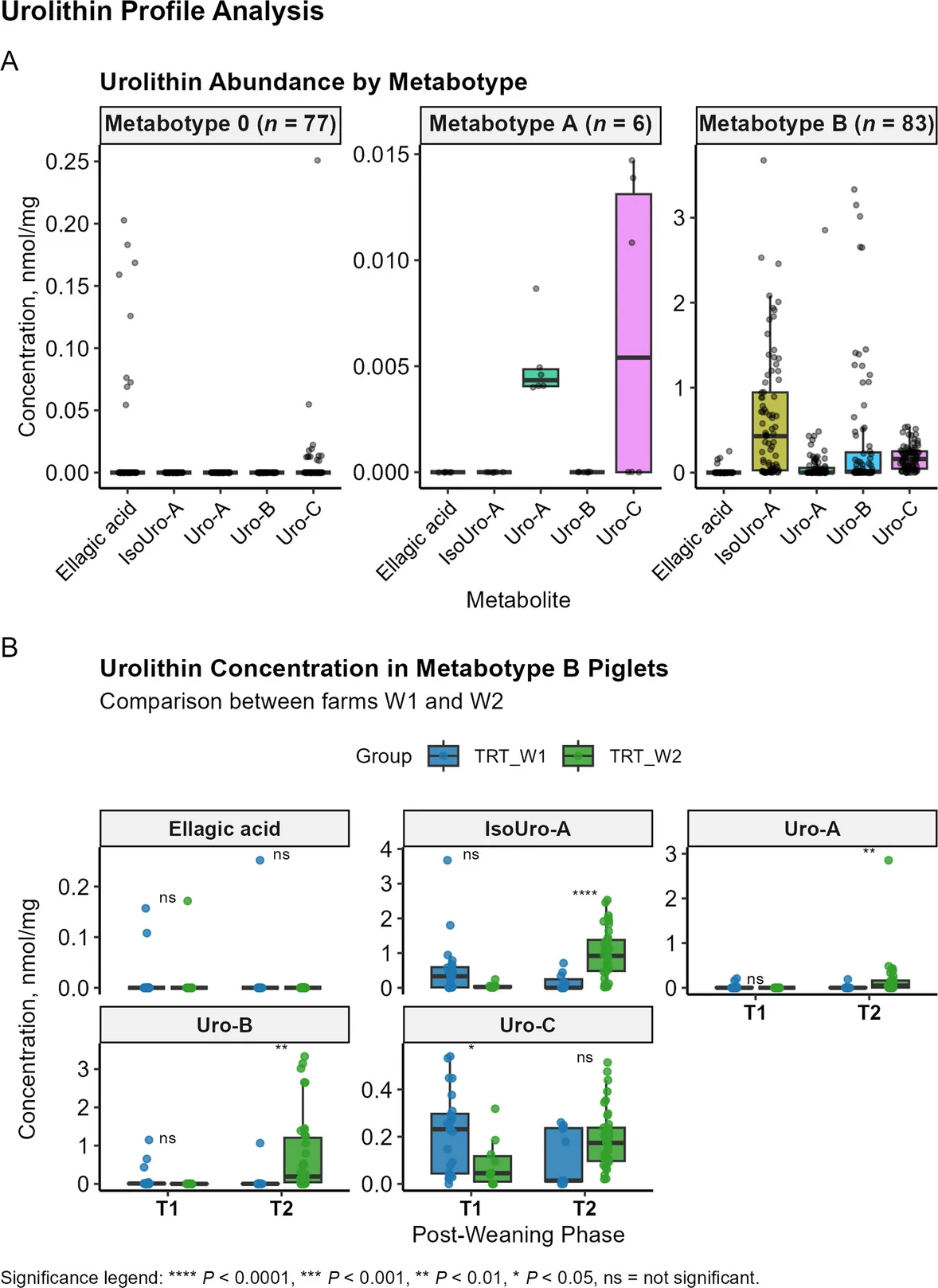
Targeted urolithin (Uro) quantification in tannin-treated piglets. **A** Distribution of Uros across metabotypes 0, A, and B based on concentration thresholds. **B** Concentrations of individual Uros in metabotype B piglets from farms W1 and W2 at T1 and T2. Significance was declared at *P* < 0.050

In the control (CTR) fecal samples analyzed in parallel (12 samples from six piglets, three per farm, at T1 and T2), none of the target metabolites—ellagic acid or urolithins—were detected. Animals were classified according to their ability to produce urolithin metabolites. Piglets were assigned to UM-B when either Uro-B or IsoUro-A was detected, to UM-A when Uro-A was detected in the absence of Uro-B and IsoUro-A, and to UM-0 when none of these metabolites were detected [38] (Fig. 5A). This classification identified the three mutually exclusive groups within the study population: UM-0 (46.4%), UM-A (3.6%), and UM-B (50.0%). Non-parametric comparisons of metabolite concentrations between TRT groups in the two farms were performed at T1 and T2 (Fig. 5B). At T1, only Uro-C differed significantly, with higher levels in TRT animals from W1 compared to W2 (*P* = 0.045). At T2, IsoUro-A (*P* < 0.001), Uro-B (*P* = 0.001), and Uro-A (*P* = 0.005) were significantly higher in TRT animals from W2 compared to TRT animals from W1. Ellagic acid did not reach statistical significance at T2 (*P* = 0.070).

The comparison between T1 and T2 revealed a clear temporal increase in urolithin-derived metabolites, particularly in TRT animals from W2. At T2, concentrations of IsoUro-A, Uro-B, and Uro-C, and Uro-A in lesser extent, were significantly higher in W2 compared with T1, suggesting an enhanced microbial biotransformation of ellagic acid over time in animals from W2. In contrast, metabolite levels in W1 appeared to remain comparatively stable or showed a less pronounced increase.

### Weighted gene co-expression network analysis

Weighted gene co-expression network analysis (WGCNA) was performed separately on species abundance profiles and KEGG Ortholog (KO) functional profiles at T2. For the species dataset (soft-thresholding power = 5), dynamic tree cutting identified six modules. Module sizes were as follows: Spec_Mod1 (*n* = 145), Spec_Mod2 (*n* = 137), Spec_Mod3 (*n* = 117), Spec_Mod4 (*n* = 109), Spec_Mod5 (*n* = 28), and Spec_Mod6 (*n* = 240). For the KO dataset (soft-thresholding power = 14). Module sizes were: KO_Mod1 (*n* = 1,248), KO_Mod2 (*n* = 683), KO_Mod3 (*n* = 407), KO_Mod4 (*n* = 254), KO_Mod5 (*n* = 150), KO_Mod6 (*n* = 138), KO_Mod7 (*n* = 117), KO_Mod8 (*n* = 112), KO_Mod9 (*n* = 22), and KO_Mod10 (*n* = 425).

**Fig. 6:**
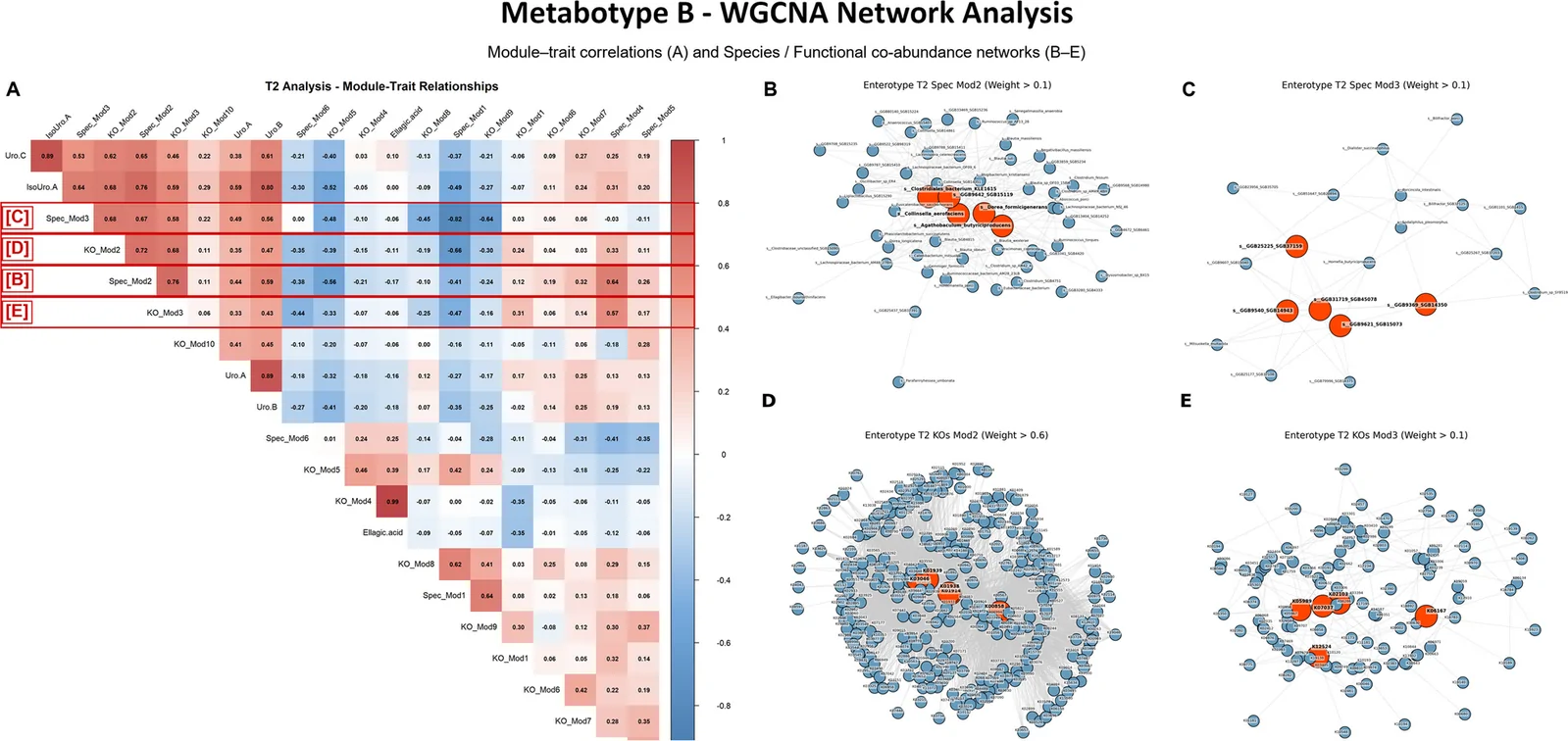
Metabotype B-specific WGCNA network analysis linking microbial community structure, functional potential, and urolithins (Uros). **A** Correlation heatmap showing associations between module eigengenes derived from species- and KO-based weighted gene co-abundance network analysis (WGCNA) and Uros. Modules with the strongest positive associations with Uros were selected for downstream network visualization. Red outlined rows and bracketed labels ([C],[D],[B],[E]) mark the module correlations shown in the correspondingly panel networs. **B** and **C** Species co-abundance networks of the selected modules (Spec_Mod2 and Spec_Mod3), highlighting the strongest TOM-based connections among taxa associated with Uro production. **D** and **E** Functional co-abundance networks of the selected KO modules (KO_Mod2 and KO_Mod3), showing the most connected KEGG Orthologs associated with the same metabolic trait. In all network panels, nodes represent microbial species or KOs, edges represent topological overlap-based co-abundance relationships, and highlighted nodes indicate hub features ranked by combined centrality

The per-animal correlation analysis between module eigengenes and individual fecal Uro concentrations, computed across UM-B piglets at T2, identified two species modules (Spec_Mod2 and Spec_Mod3) and two functional modules (KO_Mod2 and KO_Mod3) as the modules most strongly associated with Uro production (Fig. 6A). Spec_Mod2 showed strong positive correlations with IsoUro-A (*r* = 0.76), Uro-C (*r* = 0.65), Uro-B (*r* = 0.59), and Uro-A (*r* = 0.44). Similarly, Spec_Mod3 was positively correlated with IsoUro-A (*r* = 0.64), Uro-B (*r* = 0.56), Uro-C (*r* = 0.53), and Uro-A (*r* = 0.49). The KO_Mod2 correlated with IsoUro-A (*r* = 0.68), Uro-C (*r* = 0.62), Uro-B (*r* = 0.47), and Uro-A (*r* = 0.35), while KO_Mod3 correlated with IsoUro-A (*r* = 0.59), Uro-C (*r* = 0.46), Uro-B (*r* = 0.43), and Uro-A (*r* = 0.33). Importantly, strong cross-domain correlations were observed between the Uro-associated species and functional modules. Spec_Mod2 was strongly correlated with KO_Mod2 (*r* = 0.72) and KO_Mod3 (*r* = 0.76), and Spec_Mod3 showed similarly strong correlations with KO_Mod2 (*r* = 0.68) and KO_Mod3 (*r* = 0.58).

To identify the most influential features within the Uro-associated modules, hub species and KOs were obtained by filtering edges above predefined module-specific thresholds to focus on the strongest co-expression relationships, they were ranked according to a combined centrality score integrating degree, betweenness, closeness, and eigenvector centrality. The top five features for the Spec_Mod2 were *Collinsella aerofaciens* (Combined Score = 1.00), *Clostridiales bacterium* KLE1615 (0.78), *Dorea formicigenerans* (0.75), *Agathobaculum butyriciproducens* (0.73), and GGB9642_SGB15119 (0.67) (Fig. 6B). Within Spec_Mod3, the top hubs were GGB9540_SGB14943 (0.90), GGB31719_SGB45078 (0.90), GGB9621_SGB15073 (0.90), GGB25225_SGB37159 (0.77), and GGB9369_SGB14350 (0.75) (Fig. 6C). For the functional network, KO_Mod2 was driven by K01914 (Aspartate-ammonia ligase, 1.00), K01938 (Formate–tetrahydrofolate ligase, 0.98), K03046 (DNA-directed RNA polymerase subunit beta, 0.69), K01939 (Adenylosuccinate synthase, 0.68), and K00858 (NAD + kinase, 0.64), which represent the most central functional elements within this module (Fig. 6D). Similarly, KO_Mod3 was characterized by K06167 (phosphoribosyl 1,2-cyclic phosphate phosphodiesterase, 0.74), K12524 (bifunctional aspartokinase / homoserine dehydrogenase 1, 0.71), K05989 (alpha-L-rhamnosidase, 0.66), K02103 (GntR family transcriptional regulator, arabinose operon transcriptional repressor, 0.66), and K07037 (cyclic-di-AMP phosphodiesterase PgpH, 0.66) (Fig. 6E). The full list of species and KOs in these modules is provided in Additional file 4. These modules and hub features represent candidate consortia inferred from co-abundance and correlation structure; they are associative and do not establish a causal role in urolithin production.

## Discussion

In this study, we investigated the effects of supplementing weaned piglets reared in two different farms (W1 and W2) with a diet containing a chestnut–quebracho tannin blend (2 g/kg) on growth performance, diarrhea incidence, gut microbiome, and fecal metabolome. Overall, growth performance was largely unaffected by the tannin treatment: apart from a transient increase in average daily gain in tannin-fed pigs during the T1–T2 interval, no consistent differences in BW or ADG were observed between tannin-fed and control pigs in either farm, and this short-lived advantage did not persist over the whole post-weaning period. This finding is consistent with prior works showing that dietary tannins, when properly dosed, generally do not impair feed intake or growth performance in weaned piglets [9, 11]. As this was a field study with group-housed pens and ad libitum feeding, individual feed intake could not be recorded and feed conversion could not be calculated; growth was therefore assessed through individual body weight and average daily gain.

In contrast, tannin supplementation significantly reduced diarrhea occurrence in W2 during T2, whereas no diarrhea was observed in either group in the farm W1. Notably, farm W1 had experienced an early diarrhea outbreak at T1 (first week post-weaning), which led to therapeutic antibiotic administration in both dietary groups. This intervention likely masked any subsequent nutritional effect of tannins on diarrhea occurrence, as antimicrobial treatment is known to strongly suppress clinical symptoms regardless of dietary interventions. These results indicate that the efficacy of tannin supplementation may depend on the broader management and antimicrobial-use context.

This interpretation is in line with previous reports showing that tannins can reduce post-weaning diarrhea [9, 11, 39]. For example, chestnut hydrolysable tannins were shown to decrease diarrhea severity, incidence, and duration in ETEC-challenged piglets without affecting feed intake or weight gain [9]. Similarly, quebracho condensed tannins reduced diarrhea incidence and improved gut morphology, again without clear effects on growth performance [11]. However, not all studies reported beneficial outcomes, since a blend of chestnut and quebracho tannins did not impair growth but was associated with a slightly higher diarrhea incidence during the first two weeks after weaning [10]. Importantly, our work underscores that the efficacy of tannins may be more apparent in the absence of antibiotic use. This aligns with the idea that antibiotic interventions can overshadow or confound the benefits of alternative additives [40], whereas under antibiotic-free conditions tannins can act as a supportive strategy to reduce diarrhea.

To elucidate the mechanism of action of tannins we investigated the effect of tannins on the gut metagenome of pigs. Alpha-diversity of both taxonomic and functional profiles was mainly affected by farm, with lower diversity observed in farm W1 compared to W2, particularly at early post-weaning time points. This pattern is consistent with the known impact of antibiotic exposure on microbial diversity, which can impose a selective pressure on the microbial communities and reduce ecosystem complexity [41]. Interestingly, by the end of the weaning phase (T3), tannin-fed pigs in farm W2 showed a decrease in species-level alpha diversity (Shannon and Simpson) compared to controls, while functional alpha diversity remained unaffected. This might reflect a selective reshaping of microbial composition without major disruption of overall functional potential.

Beta-diversity analyses further demonstrated that tannin supplementation consistently modified the gut microbiota across all time points and farms, albeit with small effect sizes (*R*^2^ ≈ 1%–3%). Such modest effect sizes are expected for a feed additive included at a low dose (0.2%) and should not be interpreted as a weak or negligible effect: in complex gut ecosystems, large shifts in community structure (high *R*^2^) are typically driven only by major perturbations that change the substrate available to the microbiota—such as the weaning transition, antibiotic treatment, or a change in the basal diet—whereas targeted dietary additives consistently explain only a few percent of the total variation [39]. The biological relevance of the present effect is therefore supported not by significance alone but by its consistency across time points and farms and by its co-occurrence with coherent functional and health outcomes (reduced diarrhea, enrichment of beneficial taxa, and microbial urolithin production). Moreover, even where taxonomic composition diverged, the functional potential remained stable, suggesting functional redundancy whereby different taxa contribute to the same functions [42].

Differential abundance analysis highlighted farm-specific microbial responses to tannin supplementation. In farm W1, at T1, *Escherichia coli* was enriched in the control group, consistent with the diarrhea outbreak observed at that time point. In contrast, tannin-fed piglets showed lower *E. coli* abundance and, at later time points, enrichment of taxa commonly associated with fiber fermentation and short-chain fatty acid production, such as *Megasphaera elsdenii* and *Faecalibacterium prausnitzii* [43, 44]. This pattern is consistent with previous studies on similar tannin blends in weaned pigs, where supplementation promoted bacterial groups linked to gut health and microbial stability [12, 39].

In farm W2, where no comparable diarrhoeic challenge was observed, tannin supplementation was instead associated with enrichment of *Candidatus Cibionibacter* at T2 and *Lactobacillus amylovorus* at T3, indicating that tannins still modulated the microbial community, although in a different way. By contrast, the control group in W2 showed higher abundances of some *Lactobacillaceae*, including *Limosilactobacillus reuteri* and *Lactobacillus johnsonii*. Overall, these findings suggest that tannins did not exert a uniform effect across farms; rather, their impact appeared to depend on the microbial and health context, being more clearly associated with microbiota stabilization in the farm experiencing an early *E. coli*-related diarrhoeic challenge.

This farm-dependent taxonomic response was also supported at the functional level. In particular, in farm W1 the control group showed higher abundance of genes related to bacterial stress adaptation and virulence-associated functions, including hokE, which is consistent with the microbial instability and *E. coli* enrichment observed under diarrhoeic conditions. However, this result should be interpreted cautiously. The hokE encodes the toxin component of a hok/sok type I toxin-antitoxin module, classically involved in plasmid maintenance and post-segregational killing in *E. coli* [45], rather than being a canonical ETEC virulence factor by itself. Therefore, the strong abundance of hokE in CTR animals is best interpreted as evidence consistent with the expansion of plasmid-bearing *E. coli* populations in animals with poorer fecal outcomes, not as definitive proof that a specific enterotoxigenic strain was present. This interpretation is consistent with previous in vitro studies demonstrating antimicrobial activity of chestnut and quebracho tannins against enteric pathogens without complete suppression of bacterial shedding [9, 46]. Beyond this, tannin supplementation was also associated with a broader functional shift in the gut microbiome in both farms.

Beyond taxonomic and functional changes, tannin supplementation induced distinct shifts in the fecal metabolomic profile, particularly in ellagitannin-derived metabolites. Untargeted metabolomics showed that urolithins, including Uro-A, Uro-B, IsoUro-A, were among the most discriminant metabolites in tannin-fed pigs from farm W2, at later time points.

Uro-A, Uro-B, IsoUro-A are bioactive gut microbiota metabolites formed from ellagitannins and ellagic acid, known for their anti-inflammatory and barrier-protective effects [18, 47]. Their appearance in the feces of tannin treated pigs confirms that the chestnut tannins (rich in ellagitannins like castalagin/vescalagin) were indeed biotransformed by the piglet gut microbiota into urolithins. This finding provides direct evidence of microbial ellagitannin metabolism in swine, which has so far been only sparsely documented.

Moreover, these results support a potential mechanistic link between tannin supplementation and gut health outcomes. Uro-A has been shown in rodents to strengthen gut barrier function and reduce colitis severity [16, 17]. While no causal inference can be drawn from the present study, it is reasonable to hypothesize that locally produced urolithins may have contributed to the beneficial effects observed in tannin-fed piglets, including the reduction in diarrhea. Nevertheless, the antidiarrhoeic effect of tannins is unlikely to be explained by urolithin production alone. Tannins are also known to exert direct effects on the host, including astringent interactions with the intestinal mucosa, modulation of intestinal motility and secretion, and support of epithelial barrier function [3]. Beyond these direct actions, the quebracho-derived condensed tannins are themselves substrates for microbial metabolism: being poorly absorbed proanthocyanidins, they reach the hindgut largely intact and are depolymerized and catabolized by the gut microbiota into smaller phenolic metabolites—including phenyl-γ-valerolactones, phenylvaleric acids and simpler phenolic acids—that retain antioxidant, anti-inflammatory and antimicrobial activity and can further shape the microbial community [19]. Such condensed-tannin-derived catabolites may therefore have reinforced the enrichment of fiber-fermenting and butyrate-producing taxa and contributed, alongside the ellagitannin-derived urolithins, to the gut-health outcomes observed here. The present metabolomic interpretation mainly focused on ellagitannin-derived metabolites, particularly urolithins, and therefore does not provide a comprehensive characterization of the metabolic fate of the condensed tannin fraction. Although no specific flavan-3-ol-derived metabolites, such as hydroxyphenyl-γ-valerolactones, were detected, these compounds were not specifically targeted in the analytical workflow.

A puzzling aspect was why Uros were largely confined to farm W2 pigs by T2. At T1 and T2, we measured significantly higher fecal IsoUro-A, Uro-B, and Uro-A in TRT pigs of W2 compared to TRT pigs of W1 (where these metabolites remained low). The most likely explanation is the impact of antibiotic treatment in W1. As noted, all W1 pigs (CTR and TRT) received an antimicrobial treatment, whereas W2 pigs did not. Antibiotics are known to disrupt gut microbial composition and metabolism [48]; in this context, they likely impaired or eliminated key Uro-producing microbial populations in W1. Consequently, the microbial capacity to complete ellagitannin biotransformation into Uros may have been reduced in W1. Because the antimicrobial treatment was administered only in W1, the farm factor is inevitably confounded with antibiotic exposure from T2 onward, breaking the balance of the original 2 × 2 factorial design; the lower microbial diversity, reduced urolithin production and altered network structure observed in W1 after T1 therefore cannot be unambiguously attributed to intrinsic farm differences and should be interpreted with caution, so that the effect of the tannin blend is best evaluated under the antibiotic-free conditions of W2. Beyond a statistical caveat, this also illustrates how the efficacy of a feed additive can vary between farms depending on the antimicrobial and management context, since concurrent antibiotic use can suppress the very microbial converters required to bioactivate the tannins.

In contrast, a subset of pigs in W2, unexposed to antibiotics, maintained an intact microbial community capable of that conversion, although not all animals produced Uros. This inter-individual variability mirrors the human situation, in which only a subset of individuals harbors the appropriate gut microbiota to generate Uros from ellagic acid. Approximately 40% of humans efficiently produce Uro-A from ellagitannin-rich foods, while others produce mainly Uro-B (producing IsoUro-A and/or Uro-B but little Uro-A) or none at all [38]. These Uro metabotypes (UM-A, UM-B, UM-0) are quite stable within individuals and show age-related distribution patterns [38].

Interestingly, we detected only minor amounts of Uro-A in W2 pigs, whereas IsoUro-A and Uro-B predominated, suggesting that the pigs’ microbiota might preferentially support a UM-B-like profile rather than complete conversion to Uro‑A. In humans, UM-B has been associated with specific gut bacteria (e.g. some Eggerthellaceae) and is more prevalent in middle age [38]. Consistently, our targeted metabolomic analysis classified ~ 50% of the pigs as UM-B (those in TRT_W2 primarily), with only a small population as clear Uro-A producers (UM-A). These findings support the notion that individual differences in ellagitannin-conversion capacity may modulate the biological response to tannin supplementation. From an applied perspective, this suggests that tannin-based nutritional strategies may be the most effective in animals or farming systems where the relevant microbial converters are present and not suppressed by antimicrobial interventions.

To further investigate the microbial basis of Uro production, we used weighted gene co-abundance network analysis to integrate microbial taxa, functional genes, and fecal urolithin levels, restricting the analysis to piglets classified as UM-B. This approach identified two species co-abundance modules, Spec_Mod2 and Spec_Mod3, that were strongly and positively associated with fecal urolithin concentrations.

Spec_Mod2 included as central elements in the network taxa assigned to genera such as *Collinsella*, *Blautia*, and *Ruminococcus*. Within these Uro-associated modules, *Ellagibacter isourolithinifaciens* is particularly noteworthy because it is one of the few gut bacteria experimentally demonstrated to transform ellagic acid into downstream urolithins, including IsoUro-A [49]. However, its peripheral position within the network suggests that it should not be viewed as the sole driver of urolithin production, but rather as one component of a cooperative microbial network involved in sequential tannin biotransformation. This interpretation is consistent with in vitro evidence showing that complete urolithin formation can require the cooperative activity of multiple gut bacterial strains rather than a single taxon acting alone [50]. Besides, recent in vivo evidence further supports this concept, as the oral administration of defined bacterial consortia enriched in Uro-associated taxa, such as *Gordonibacter* and *Enterocloster* (consortium A) or *Ellagibacter* and *Enterocloster* (consortium B), has been shown to successfully transfer the Uro-producing phenotype to previously non-producing hosts, demonstrating that coordinated colonization by multiple taxa can establish functional Uro metabolism within the gut ecosystem [51]. In the present study, however, the association between these consortia and urolithin production remains correlative: definitive attribution would require functional verification, such as in vitro fermentation of ellagitannins with defined microbial communities or controlled colonization of gnotobiotic animals, which represents the natural next step rather than evidence provided here.

A similar interpretation may apply to the *Lachnospiraceae*-related species detected in the same network. Members of the *Lachnospiraceae* have been associated with urolithin-producing microbiota, particularly the UM-A metabotype, and more recent mechanistic evidence indicates that some taxa within this broader lineage can catalyze downstream dehydroxylation steps in the conversion of Uro‑C to Uro‑A [14, 52]. Thus, while Eggerthellaceae-related bacteria such as *Ellagibacter* are the best-supported candidates for the early steps of ellagitannin transformation, *Lachnospiraceae* may contribute to later reactions, collectively enabling completion of the metabolic pathway. The co-occurrence of these taxa within the same modules therefore supports a division-of-labor model for urolithin biosynthesis.

Notably, the network analysis also highlighted several hub taxa that are unlikely to be direct urolithin producers but could contribute through metabolite cross-feeding or ecological stabilization of the urolithin-producing community. In particular, several butyrate-producing bacteria, including *Faecalibacterium*, *Butyrivibrio*, and *Pseudobutyrivibrio*, consistently co-occurred with higher urolithin levels. Rather than directly catalyzing ellagitannin conversion, these organisms may benefit from the altered intestinal environment generated by tannin degradation, for example through reduced proteolytic fermentation, lower pathogen pressure, or increased availability of fermentable substrates released by primary degraders [53]. In turn, their production of butyrate could reinforce epithelial energy metabolism and gut barrier integrity, thereby functionally complementing the anti-inflammatory and barrier-supporting effects attributed to urolithins [17, 18]. Taken together, these findings argue against a single “urolithin-producer” model and instead support a consortium-based framework, in which tannin metabolism is distributed across functionally distinct but metabolically connected taxa: primary ellagitannin converters, secondary urolithin-modifying bacteria, and butyrate-producing community members that contribute to ecosystem stability [14].

Supporting this interpretation, the WGCNA identified two KO modules associated with fecal urolithins. KO_Mod2 was mainly composed of housekeeping functions involved in nucleotide and amino acid biosynthesis, translation, and DNA metabolism, suggesting that this module reflects the overall metabolic activity of the urolithin-associated community rather than a pathway-specific signature. This indicates that urolithin production is linked to an active and metabolically engaged microbial consortium, rather than to the enrichment of a single dedicated pathway. In contrast, KO_Mod3 contained functions more plausibly linked to the processing of complex dietary substrates, including alpha-L-rhamnosidase (K05989), beta-glucosidase (K05350), and other carbohydrate-active transport and regulatory genes. Alpha-L-rhamnosidases cleave terminal rhamnose residues from plant glycosides and are commonly involved in the initial microbial deconjugation steps of dietary polyphenols. Thus, K05989 is unlikely to represent the core catalytic step of urolithin formation itself but may instead mark a broader glycoside-degrading microbial consortium that facilitates access to tannin-derived substrates and supports cross-feeding interactions within the Uro-producing microbiota. This interpretation is consistent with recent studies showing that the direct downstream conversion of urolithin intermediates relies on specialized gut bacterial dehydroxylases, while several additional steps in the ellagitannin-to-Uro pathway remain incompletely annotated [54–57].

## Conclusions

Supplementation of weaned piglet diets with a chestnut–quebracho tannin blend (2 g/kg) did not impair growth performance but possibly reduced the prevalence of post-weaning diarrhea under antibiotic-free conditions. Metagenomics and Metabolomics analyses showed that tannins induced consistent shifts in gut microbial composition while largely preserving functional potential, suggesting that tannins act by selectively reshaping the microbiota rather than broadly disrupting its metabolic capacity. Notably, tannin supplementation was associated with the microbial production of ellagitannin-derived metabolites, particularly urolithins, mainly in pigs not treated with antibiotics, indicating active microbial biotransformation of dietary polyphenols. Network-based integration of metagenomic and metabolomic data further suggested that urolithin production is driven by cooperative microbial consortia rather than single specialized taxa. Accordingly, inter-individual and inter-farm variability in microbial tannin biotransformation capacity likely contributes to the heterogeneous responses to tannin supplementation.

Together, these results support tannins as promising microbiota-modulating nutritional tools that may help reduce reliance on antibiotics when microbial functionality is preserved. Future research should explore how microbiome composition influences tannin responsiveness, whether microbial interventions could enhance urolithin-producing capacity, and how dietary tannin strategies can be optimized to promote beneficial microbial metabolism and long-term gut resilience in pigs.

## Supplementary Information

Additional file 1. Ingredient composition and calculated nutrient content of the basal diets used in the three feeding phases.

Additional file 2. Discriminant species identified by linear discriminant analysis effect size between experimental groups at T1, T2 and T3, with LDA scores, raw and adjusted *P* values, the enriched group, and the nearest resolved taxonomic annotation for unresolved MetaPhlAn genome bins.

Additional file 3. Significant KEGG Orthologs identified by differential abundance analysis between control and tannin-treated piglets at T1, T2 and T3 in farms W1 and W2.

Additional file 4. Full list of species and KEGG Orthologsincluded in the WGCNA modules associated with fecal urolithin concentrations.

## Data Availability

The shotgun metagenomic sequencing data generated and analyzed in this study have been deposited in the European Nucleotide Archive under accession number PRJEB80533.

## Abbreviations

ADG: Average daily gain
BW: Body weight
CT: Condensed tannins
CTR: Control diet
DM: Dry matter
EA: Ellagic acid
ETEC: Enterotoxigenic *Escherichia coli*
HT: Hydrolysable tannins
KEGG: Kyoto Encyclopedia of Genes and Genomes
KO: KEGG Orthology
LEfSe: Linear discriminant analysis Effect Size
LDA: Linear discriminant analysis
PCoA: Principal coordinate analysis
PERMANOVA: Permutational multivariate analysis of variance
PWD: Post-weaning diarrhea
QC: Quality control
SEM: Standard error of the mean
TRT: Tannin-supplemented diet
UM: Urolithin metabotype
Uro: Urolithin
Uro-A: Urolithin A
Uro-B: Urolithin B
IsoUro-A: Isourolithin A
UPLC-MS/MS: Ultra-performance liquid chromatography tandem mass spectrometry
WGCNA: Weighted gene co-expression network analysis
