# Supplementary material for: Chestnut–quebracho tannins shape the fecal metabolome of weaned pigs through microbiota-dependent urolithin production
Source: J Anim Sci Biotechnol. 2026 Oct 6;17:188. doi: 10.1186/s40104-026-01503-x (PMC13640353; doi:10.1186/s40104-026-01503-x)
Supplement: Supplementary file 1 [file 40104_2026_1503_MOESM1_ESM.docx]

| Item | Phase 1 (d0-35) | Phase 2 (d36-49) | Phase 3 (d50-76) |
| --- | --- | --- | --- |
| Ingredients, % as-fed |  |  |  |
| Barley | 23.28 | 26.11 | 20.00 |
| Corn | 5.00 | 5.00 | 20.24 |
| Wheat | 14.00 | 16.00 | 14.00 |
| Cooked corn and barley | 12.00 | 12.00 | 10.00 |
| Soybean meal, dehulled, solvent-extracted | — | — | 10.00 |
| Biscuits | 9.00 | 8.00 | 5.00 |
| Full-fat soybean | 5.50 | 6.50 | 4.00 |
| Sweet whey powder | 6.50 | 6.00 | — |
| Oat bran | 5.50 | 5.00 | — |
| Fish meal | 2.50 | 4.50 | — |
| Fermented milk product | 4.00 | — | — |
| Soybean protein concentrate | 3.00 | 4.00 | 4.00 |
| Sugar beet pulp | — | — | 4.00 |
| Animal fat | — | — | 2.60 |
| Porcine plasma | 2.50 | — | — |
| Maltodextrin | 1.50 | 1.00 | — |
| Piglet vitamin-mineral premix | 1.00 | 1.00 | 0.70 |
| Coconut oil | 1.00 | 1.00 | — |
| Salmon oil | 1.00 | 1.00 | — |
| Lignocellulose | 0.80 | 0.80 | 0.80 |
| Organic acids | — | — | 0.80 |
| L-Lysine 78% | 0.58 | 0.62 | 0.60 |
| Citric acid | — | — | 0.60 |
| Dicalcium phosphate | — | — | 0.60 |
| Limestone | — | — | 0.50 |
| Sodium chloride | 0.10 | 0.25 | 0.45 |
| L-Threonine | 0.26 | 0.26 | 0.25 |
| DL-Methionine | 0.24 | 0.24 | 0.24 |
| Nutri P (tannin blend)* | 0.20 | 0.20 | 0.20 |
| L-Valine | 0.12 | 0.13 | 0.10 |
| Calcium butyrate | 0.10 | 0.08 | 0.10 |
| *Bacillus subtilis* + *Bacillus licheniformis* | 0.10 | 0.08 | 0.05 |
| L-Tryptophan | 0.08 | 0.10 | 0.07 |
| Choline chloride 75% | 0.08 | 0.08 | 0.07 |
| Flavours | 0.06 | 0.05 | 0.03 |
| Calculated nutrient content |  |  |  |
| Moisture, % | 10.34 | 10.72 | 11.37 |
| Dry matter, % | 89.66 | 89.28 | 88.63 |
| ME, kcal/kg | 3254.37 | 3221.12 | 3245.34 |
| NE, kcal/kg | 2390.47 | 2360.74 | 2368.97 |
| Crude protein, % | 17.17 | 16.26 | 15.75 |
| Crude fat, % | 5.60 | 5.71 | 5.25 |
| Crude fiber, % | 4.68 | 4.77 | 4.53 |
| Crude ash, % | 3.59 | 3.60 | 4.11 |
| Calcium, % | 0.35 | 0.33 | 0.47 |
| Phosphorus, % | 0.43 | 0.43 | 0.45 |
| Sodium, % | 0.28 | 0.25 | 0.22 |
| Chloride, % | 0.48 | 0.54 | 0.48 |
| Potassium, % | 0.66 | 0.68 | 0.67 |
| Magnesium, % | 0.15 | 0.15 | — |
| Sugars, % | 11.03 | 9.30 | 4.54 |
| Starch, % | 35.19 | 37.29 | 40.40 |
| Lactose, % | 5.54 | 4.03 | — |
| Total methionine, % | 0.49 | 0.49 | 0.45 |
| TSAA, % | 0.80 | 0.75 | 0.71 |
| Total lysine, % | 1.31 | 1.25 | 1.19 |
| Total threonine, % | 0.88 | 0.81 | 0.77 |
| Total valine, % | 0.93 | 0.84 | 0.79 |
| Total arginine, % | 0.88 | 0.84 | 0.91 |
| Isoleucine, % | 0.64 | 0.60 | 0.59 |
| Total leucine, % | 1.22 | 1.11 | 1.13 |
| Total tryptophan, % | 0.28 | 0.28 | 0.24 |
| Total phenylalanine, % | 0.75 | 0.68 | 0.71 |
| Histidine, % | 0.39 | 0.35 | 0.36 |
| Total glycine, % | 0.64 | 0.67 | 0.60 |
| Total tyrosine, % | 0.49 | 0.42 | 0.39 |
| SID methionine, % | 0.45 | 0.46 | 0.42 |
| SID TSAA, % | 0.72 | 0.68 | 0.63 |
| SID lysine, % | 1.20 | 1.13 | 1.08 |
| SID threonine, % | 0.78 | 0.72 | 0.67 |
| SID valine, % | 0.81 | 0.73 | 0.68 |
| SID arginine, % | 0.80 | 0.76 | 0.80 |
| SID tryptophan, % | 0.26 | 0.25 | 0.21 |
| SID isoleucine, % | 0.53 | 0.50 | 0.50 |
| SID leucine, % | 1.03 | 0.92 | 0.96 |
| SID histidine, % | 0.34 | 0.31 | 0.32 |
| SID phenylalanine, % | 0.53 | 0.44 | 0.46 |

**Additional file 1. Ingredient composition and calculated nutrient content of the basal diets used in the three feeding phases (Phase 1: d 0–35; Phase 2: d 36–49; Phase 3: d 50–76).**

Values are expressed on an as-fed basis unless otherwise stated. ME, metabolizable energy; NE, net energy; TSAA, total sulfur amino acids; SID, standardized ileal digestible.
* **Nutri P** is a blend of **condensed and hydrolysable tannins (Silvafeed®Nutri P, Silvateam, Italy).**
**Premix composition (per kg of premix):** vitamin A, 3,200,000 IU; vitamin D3, 400,000 IU; vitamin E, 15,000 mg; vitamin B1, 600 mg; vitamin B2, 1,200 mg; vitamin B6, 800 mg; vitamin B12, 10 mg; niacin, 16,000 mg; vitamin C, 25,000 mg; vitamin K3, 590 mg; folic acid, 770 mg; biotin, 60 mg; pantothenic acid, 4,200 mg; Zn, 20,600 mg; Mn, 6,400 mg; Cu, 25,000 mg; Fe, 15,000 mg; I, 620 mg; Se, 60 mg; xylanase, 432,000 EPU; 6-phytase, 200,000 FYT.
